# Current oral hygiene and recreational behavioral trends in HIV disease

**DOI:** 10.1002/cre2.762

**Published:** 2023-07-04

**Authors:** Donald E. Mercante, Emily Guarisco, Elizabeth A. Lilly, Arni Rao, Kelly Treas, Clifford J. Beall, Zach Thompson, Ann L. Griffen, Eugene J. Leys, Jose A. Vazquez, Michael E. Hagensee, Paul L. Fidel

**Affiliations:** ^1^ Department of Biostatistics, Biostatistics Program, School of Public Health Louisiana State University Health New Orleans Louisiana USA; ^2^ Department of Oral and Craniofacial Biology, Louisiana State University Health School of Dentistry New Orleans Louisiana USA; ^3^ Division of Infectious Diseases, Department of Medicine, School of Medicine, Medical College of Georgia Augusta University Augusta Georgia USA; ^4^ Department of Microbiology and Molecular Genetics, School of Dentistry The Ohio State University Columbus Ohio USA; ^5^ Department of Pediatric Dentistry, School of Dentistry The Ohio State University Columbus Ohio USA; ^6^ Section of Infectious Diseases, Department of Medicine, School of Medicine Louisiana State University Health New Orleans LA USA

**Keywords:** epidemiology, HIV disease, oral hygiene, people living with HIV, recreational behaviors

## Abstract

**Objective:**

HIV disease is evolving with more HIV+ persons experiencing a high quality of life with well‐controlled viremia. We recently enrolled a large cohort of HIV+ and clinically relevant HIV− persons for oral microbiome analyses that included a questionnaire related to oral hygiene and recreational behaviors. Here, the questionnaire responses were analyzed for behavioral trends within the cohort, together with trends over time by comparison to a previous geographically centered HIV+ cohort.

**Methods:**

Data were collected by questionnaire at baseline visits as cross‐sectional assessments. Multivariable analyses were conducted for associations of HIV status as well as age, race, and sex, on oral hygiene/recreational behaviors.

**Results:**

HIV+ subjects had reduced brushing frequency, but increased incidence of past cleanings and frequency of dry mouth, compared to the HIV− subjects. Within the entire cohort, positive associations were identified between age and several oral hygiene practices, and between age, race, and sex for several recreational behaviors. In comparison to the historical cohort, the contemporary HIV+ cohort participated in fewer high‐risk behaviors, but with similar trends for smoking and oral hygiene practices.

**Conclusion:**

HIV status had little association with oral hygiene and recreational behaviors despite several differences in age, race, and sex. Behavioral trends over time support a higher quality of life in people currently living with HIV.

## 1 INTRODUCTION

The epidemiologic risk factors that lead to HIV infection have been a topic for many years. Most studies show persons living with HIV (PLWH) to have high‐risk social behaviors. These include current smoking (42%–54%), heavy alcohol use (15%–19%, as defined by >4 drinks per day), and illicit drug use other than marijuana (21%–40%). In contrast, the HIV− subjects in the cohorts (mostly individuals at low risk for HIV exposure) have significantly lower rates of current smoking (18%–20%), heavy alcohol use (7%–10%), and drug use (3%–8%). However, these studies had cohorts that were recruited before 2015 and may not reflect current behaviors in PLWH (Bien‐Gund et al., 2021; Bing et al., 2001; Cook et al., 2015; Dawson‐Rose et al., 2017; Mahon, 1996; Mdodo et al., 2015; Pacek & Cioe, 2015; Park et al., 2016; Skalski et al., 2015; Washington et al., 2013).

Stronger therapeutic antiretroviral drug regimens (ART) have led to a longer lifespan and improved quality of life; therefore, it is likely that behaviors have changed as well. A recent clinical study was conducted to examine the differences in the oral microbiome (bacteriome and mycobiome) between HIV+ and HIV− persons (Fidel et al., 2021; Griffen et al., 2019). The cohort used in the microbiome analyses consisted of 341 subjects, including 89 HIV− subjects and 252 HIV+ subjects all on ART enrolled from 2013 to 2018. Also enrolled were HIV+ persons not on ART who were followed longitudinally after initiation of ART. Enrollment for the primary study design attempted to match clinical variables as much as possible with HIV− persons possessing high‐risk behavior for HIV exposure (often enrolled at sexually transmitted disease clinics). Baseline demographic and behavioral data (questionnaire) was collected on all participants and an oral exam assessed periodontal disease, caries, and prevalence of oropharyngeal candidiasis (OPC) and human papillomavirus (HPV)‐associated oral warts or cancers. Our previous report, which included initial unadjusted statistical analyses on the demographic data or questionnaire responses, showed several differences between HIV+ and HIV− persons including race, oral sex encounters, illicit drug use, oral hygiene practices, antibiotic use, and antifungal use. Differences in continuous variables from demographic data or oral exam included reduced incidence of gingivitis, an increased number of missing teeth, and age (Griffen et al., 2019). No differences were noted in the other variables, including sex, alcoholic beverage consumption, diet, nonalcoholic beverage consumption, oral yeast (*Candida albicans)* carriage, marijuana use, periodontal disease, caries status, and caries restorations (Griffen et al., 2019). The purpose of this study was to evaluate the full range of baseline visit responses for oral hygiene and recreational behaviors in multivariable analyses using the entire cohort adjusted for age, race, and sex to identify differences based on HIV status. In addition, we also tested the hypothesis that many behaviors likely changed over time by comparing the similar parameters between PLWH in the contemporary cohort and those from a previous geographically centered HIV+ cohort (1998–2004) (Leigh et al., 2006; McNulty et al., 2005; Mercante et al., 2006; Quimby et al., 2012).

## 2 METHODS

### 2.1 Cohorts and study design

This study consisted of two cohorts of HIV+ and HIV− subjects that were evaluated as part of two independent NIH‐funded grants conducted at the Louisiana State University Health Sciences Center School of Dentistry. Both studies were conducted in accordance with the Institutional Review Board (IRB) at the Louisiana State University Health Sciences Center—New Orleans (LSUHSC‐NO) (IRB #3193). The contemporary cohort included 398 HIV+ individuals and 100 HIV− individuals enrolled between 2013 and 2018, all of whom completed a questionnaire surveying oral hygiene and recreational behaviors as part of a primary experimental design evaluating the oral microbiome. The questionnaire collected data on 26 categorical and continuous topics at the baseline visit. This study included the analysis for those questions pertaining to oral hygiene and recreational behaviors (*n* = 14). Each question had multinomial responses. The analysis between HIV+ and HIV− persons in the cohort was cross‐sectional. The prior cohort consisted of 193 HIV+ and 97 HIV− individuals enrolled between 1998 and 2004 as part of a primary experimental design to evaluate the immune parameters of OPC (Mercante et al., 2006; Quimby et al., 2012). Data were similarly collected by questionnaire at enrollment (baseline visit). In both cohorts, attempts were made to enroll HIV− individuals who were at high risk to acquire HIV to provide a clinically relevant control group (most enrolled at sexually transmitted disease clinics). The analysis between the contemporary and previous cohorts was cross‐sectional in nature and limited to data from only the HIV+ subjects in an effort to determine how behavioral trends have changed in the HIV+ population over time. The sample size in each cohort was based on power analyses related to the primary scientific design of each study. Finally, access to free dental care was equal in both HIV+ cohorts with services housed in the same building as primary care. Results and data were reported/illustrated according to the STROBE guidelines (Vandenbroucke et al., 2007).

### 2.2 Statistical analyses

Age was used as either a continuous predictor or as a categorical predictor variable by stratifying individuals into three age groups: ≤30, 31–50, and ≥51 years old. Race was stratified as African American (Black), Caucasian (White), and Others. Initial unadjusted analyses assessing associations between HIV status and demographic and behavioral variables were performed using either *χ* ^2^ tests or Fisher's exact test for categorical variables and *t* tests for continuous variables. Although all participants are included in unadjusted analyses, individuals in the “Other” race category were not included in the multivariable models due to the limited number of subjects.

General linear models were fit to test for associations between age group, race, sex, and continuous recreational behaviors, such as smoking duration and amount. These models also included HIV status as a main effect. The rationale for the covariates included in the models pertained to the focus on oral health and recreational behaviors associated with risk for HIV exposure. Categorical outcomes with binary responses were analyzed with binary logistic regression (logit) models with binomial error distribution. Outcomes with ordinal categorical response profiles were analyzed with cumulative logit models. Cochran–Mantel–Haenszel test was used for assessing the linear association between smoking status and frequency of alcohol use, adjusting for HIV status. The Statistical Analysis System (SAS) Version 9.4 was used for all analyses. PROC GLM was used to fit general linear models involving continuous outcomes and mixed scale predictors/covariates. PROCs LOGISTIC and GENMOD were used for fitting the binary and cumulative logit models. PROCs TTEST and UNIVARIATE were used for comparing means or medians between two independent samples. PROCs MEANS and FREQ were used to generate descriptive statistics.

Regression coefficient estimates, adjusted odds ratios (aOR), associated *p* values, and confidence intervals were obtained for testing and estimation of associations between behavior variables and HIV status, age, race, and sex. The CONTRAST statement in PROC LOGISTIC or GENMOD was used to compare levels of categorical variables not involving the reference group. For continuous variables involving cigarette smoking behaviors (smoking duration, packs/day), general linear models and appropriate linear contrasts were performed to determine differences in the amount or length of the smoking behaviors as a function of age, race, and sex. Although each multivariable model included HIV status, age, race, and sex as independent factors, associations with HIV status are presented separately.

For comparisons between the two sets of cohorts (1998–2004) and (2013–2018), the proportion of behaviors for HIV+ persons as a function of age, race, and sex were modeled using logistic regression models. Blood CD4 cell counts were compared using both the Student's *t* test (mean) or Wilcoxon's Rank Sum test (median) due to possible skewness in the distribution. Of note, the 1998–2004 cohort consisted of two studies, a cross‐sectional study consisting of 158 HIV+ subjects at the baseline visit, and a longitudinal study of 35 HIV+ subjects at the baseline visit.

## 3 RESULTS

The demographic data for the contemporary cohort (2013–2018) are summarized in Table 1 for both PLWH (*n* = 398) and HIV− (*n* = 100) persons at high risk for HIV exposure. Notably, the PLWH group had a higher average age compared to the HIV− group (47 vs. 40 years).

**Table 1:** Demographic characteristics of study participants in the contemporary cohort.

Discrete variables	HIV+		HIV−	
	*N*	Percent	*N*	Percent
	398	79.9	100	20.1
Age group				
18–30	52	13.1	31	31.0
31–50	150	37.7	43	43.0
>50	196	49.2	26	26.0
Sex				
Female	115	28.9	30	30.0
Male	283	71.1	70	70.0
Race/ethnicity				
White	73	18.3	35	35.0
Black	306	76.9	61	61.0
Other	19	4.8	4	4.0
Ever a smoker	275	69.8	76	76.0
Current smoker	196	49.8	57	57.6
Alcohol use	207	52.1	41	41.4
Daily brushing	353	88.9	97	97.0
Never brush	24	6.1	1	1.0
Daily flossing	138	35.5	30	31.3
Daily mouthwash use	229	77.9	44	69.8
Frequent dry mouth	118	30.2	18	18.6
Oral sex	111	28.4	40	40.0
Illicit drug use	23	27.4	40	53.3
Marijuana	15	14.9	28	36.4
Diet				
Typical	247	64.2	58	58.6
Other	112	29.2	36	36.4
Unknown	25	6.5	5	5.1
Carbonated drinks	286	71.9	79	79.0
OPC	44	11.1	0	0
Oral warts	8	2.0	1	1.0
Continuous variables				

	Mean	SD	Mean	SD
Age	47.4	12.0	39.6	12.7
Years smoked	21.6	12.1	17.0	10.2

Abbreviation: OPC, oropharyngeal candidiasis.

**Figure 1:**
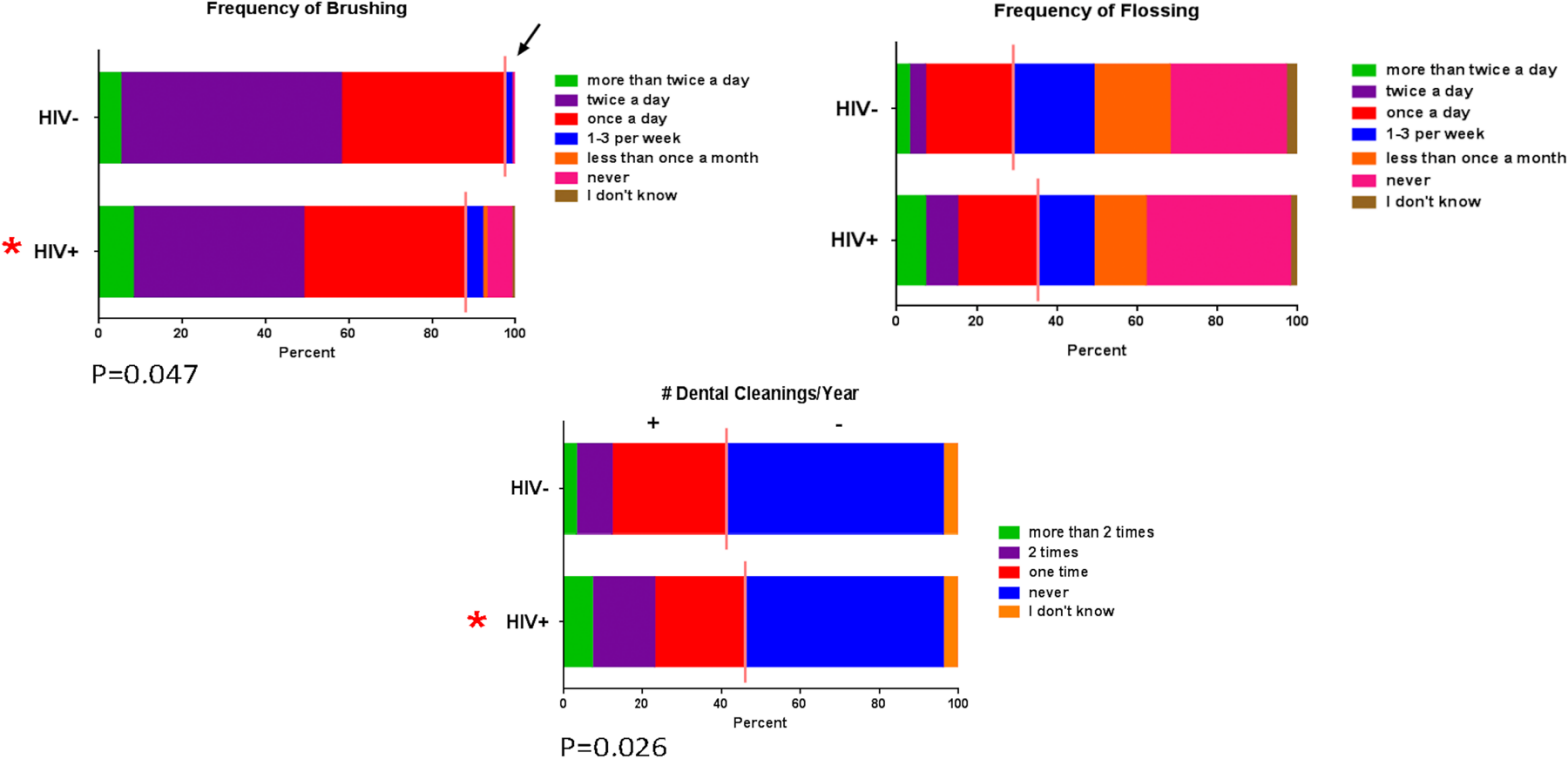
Oral hygiene behavior responses. The breakpoints from the initial analysis are shown by the solid lines. Further breakdown of the individual responses shown by the various color‐coded bars. *, Statistical differences in the initial analysis.

**Figure 2:**
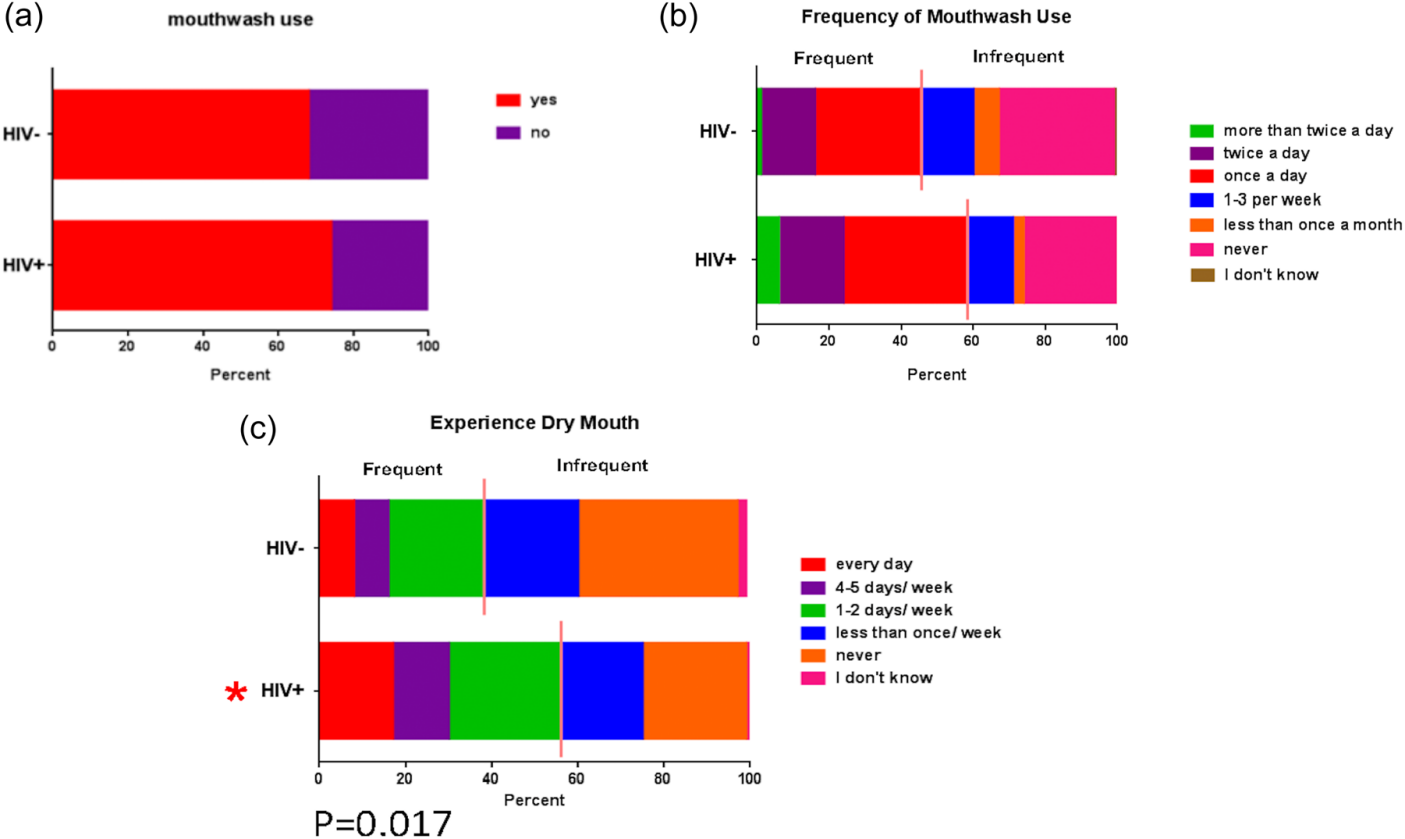
Responses for mouthwash use and dry mouth. (a) mouthwash use; (b) frequency of mouthwash use; (c) experience dry mouth. *, Statistical differences in the generalized analysis. The generalized analysis stratified frequency of mouthwash use as frequent or infrequent, shown by the solid line.

HIV+ individuals brushed less frequently than HIV− individuals [aOR = 3.56 (1.02,12.49)], flossed similarly to HIV− subjects, and reported a higher frequency of past cleanings as compared with HIV− persons [aOR = 1.68 (1.06, 2.66)] (Figure 1). The frequency of having at least two cleanings in the past year was higher in HIV+ persons [aOR=2.71 (1.23, 5.99), *p* = .013)]. There was no association regarding HIV status for mouthwash use or frequency of mouthwash use. In a separate model evaluating the frequency of xerostomia (dry mouth), the adjusted analysis also showed HIV+ persons experience xerostomia more frequently when compared to HIV− persons [aOR = 2.06 (1.14, 3.71)] (Figure 2).

**Figure 3:**
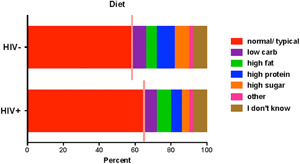
Dietary responses. The generalized analysis stratified diet by typical versus other, shown by the solid line.

Most persons were categorized as having a “typical” diet (63.2%), with the remainder categorized as either “other than typical” (30.6%) or “unknown” (6.2%). A multiple logistic model found no associations among any of the covariates, HIV status, age, gender or race, and diet (Figure 3).

HIV− persons reported greater consumption of nonalcoholic beverages when compared to HIV+ persons. Specifically, HIV− persons consumed carbonated drinks much more frequently than HIV+ persons [aOR = 1.90 (1.05, 3.44), *p* = .033)] as well as coffee [aOR = 2.25 (1.34, 3.80), *p* = .002)] and sports drinks [aOR = 1.68 (1.04, 2.71), *p* = .033].

**Figure 4:**
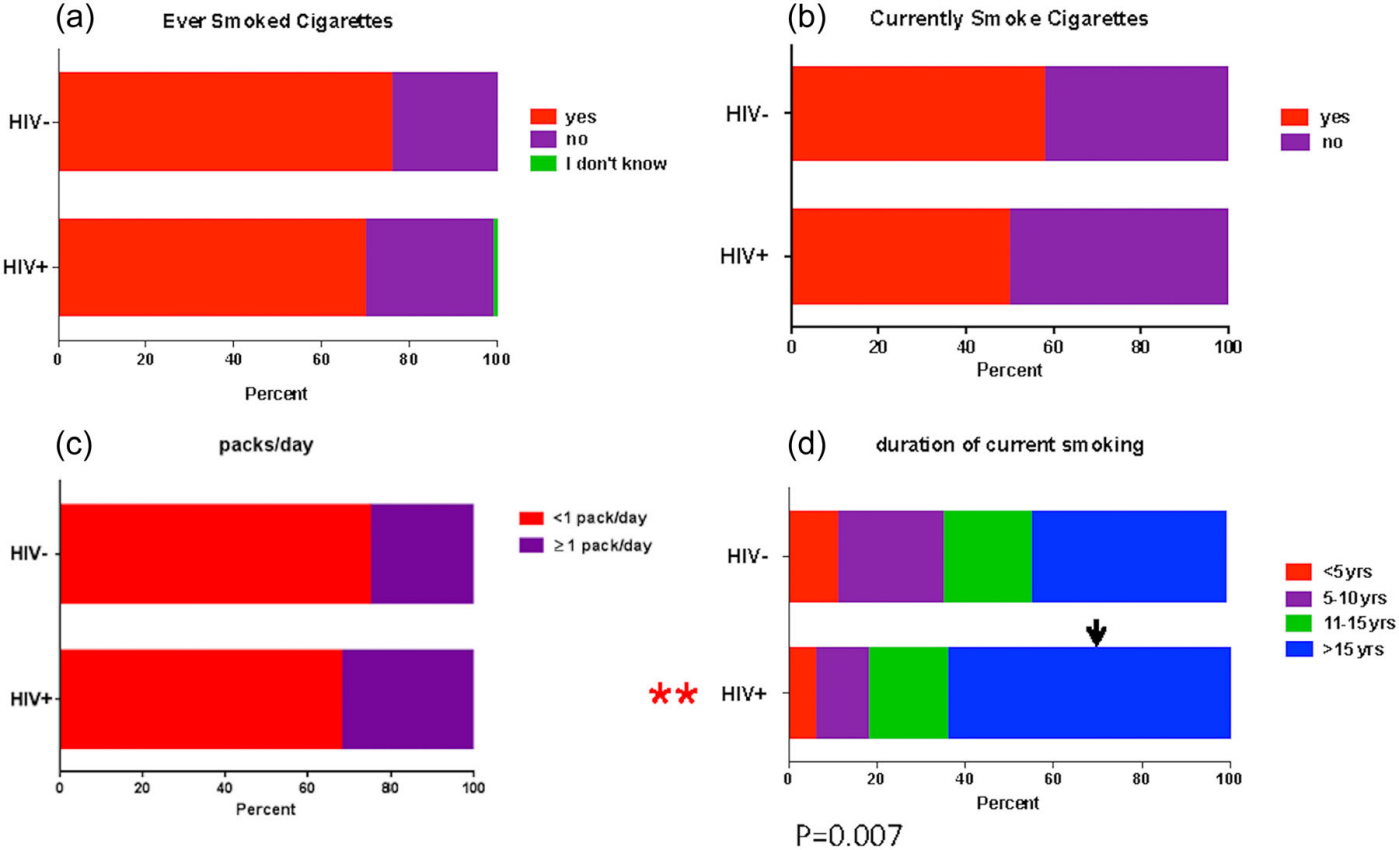
Responses for cigarette smoking history, duration, and frequency. (a) ever smoked cigarettes; (b) currently smoke cigarettes; (c) packs/day; (d) duration of current smoking. **, Statistical differences in the generalized analysis.

HIV status was not associated with cigarette smoking, both for current smokers or in the frequency of packs smoked/day. However, HIV status did have an impact on the duration of smoking in the current smokers (Figure 4).

**Figure 5:**
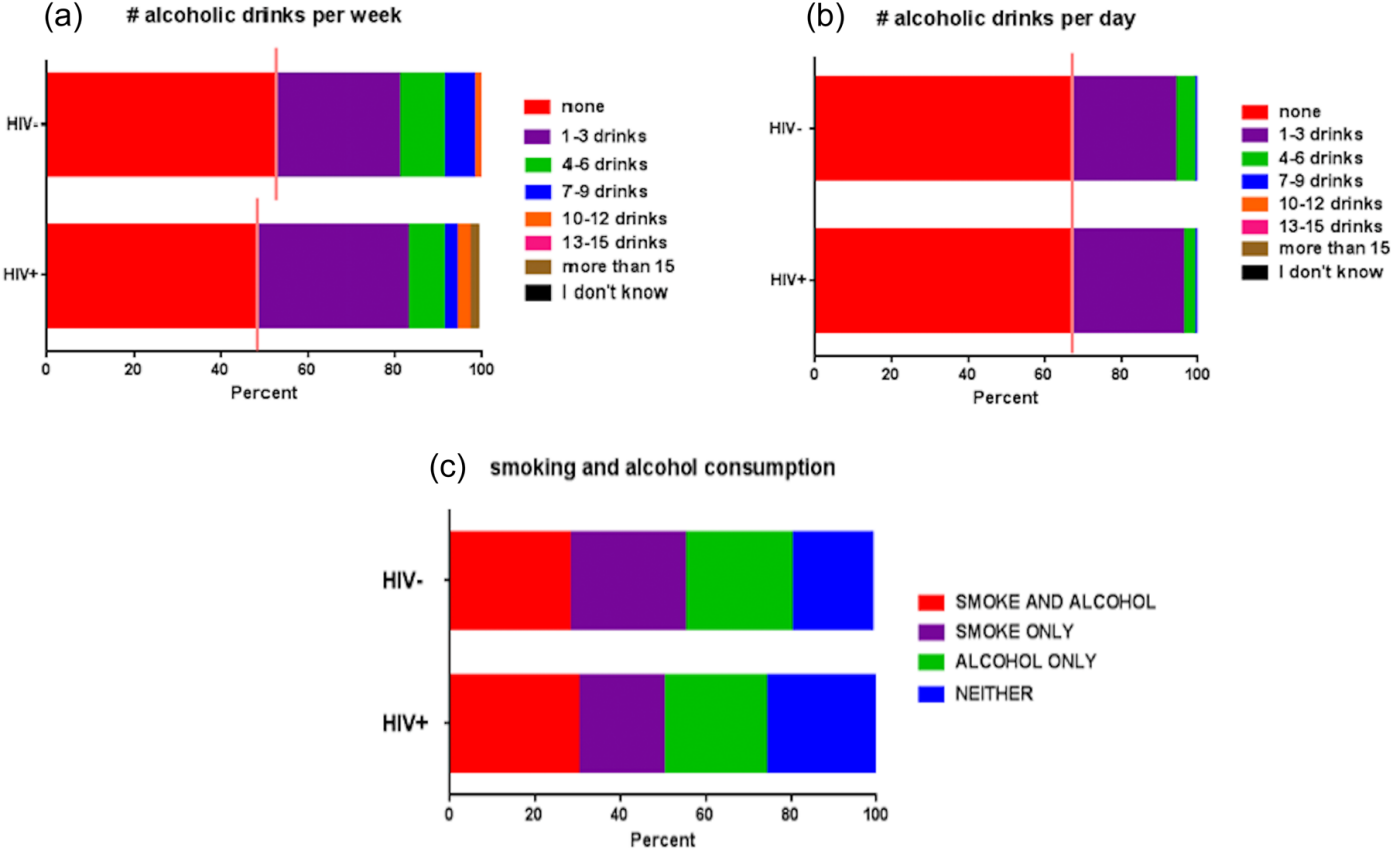
Responses for alcoholic beverage consumption. The generalized analysis stratified alcoholic beverage consumption by none versus one or more, shown by the solid line. (a) # alcoholic drinks per week; (b) # alcoholic drinks per day; (c) smoking and alcohol consumption.

There was no effect of HIV status on either daily or weekly alcohol consumption. On the other hand, alcohol use was positively associated with smoking (*p* = .0005) with smokers more likely to use alcohol than nonsmokers [aOR = 2.11 (1.41, 3.17), *p* = .0003)] (Figure 5).

Table 2 shows the comparative results for multivariable analyses of the various behaviors on the entire cohorts. When brushing frequency was compared to the older age group (>50 years), individuals 31–50 and ≤30 years of age brushed more frequently, respectively), irrespective of HIV status. A positive association was also found between the frequency of brushing and flossing (*p* = .0005). For flossing frequency, individuals 31–50 years and those >50 years flossed more than individuals ≤30 years of age. Additionally, persons 31–50 years old and persons >50 years old had higher proportions of at least two prior cleanings in the past year as compared to those ≤30 years old. Regarding mouthwash use, although there were no differences among the different age cohorts or sex, Black persons utilized mouthwash more frequently than White persons. In addition, individuals reporting greater mouthwash use also had higher frequencies of daily brushing reported (*p* = .0018). Xerostomia was reported more frequently by females than by males.

**Table 2:** Multivariable analyses for the full contemporary cohort of HIV+ and HIV− subjects.

	Age group		Sex		Race	
Outcome	Comparison^a^	aOR (lb, ub); *p* a	Comparison	aOR (lb, ub); *p*	Comparison	aOR (lb, ub); *p*
Daily brushing^a^	>50 vs. <30	12.64 (1.66, 96.41); **.014**	Male vs. Female	0.93 (0.69, 2.01); .960	White vs. Black	0.66 (0.31, 1.41); .288
	>50 vs. 31–50	2.08 (1.02, 4.23); **.044**				
Flossing frequency	31–50 vs. <30	1.97 (1.04, 3.74); **.038**	Male vs. Female	1.22 (0.78, 1.93); .386	White vs. Black	1.43 (0.86, 2.37); .1645
	>50 vs. <30	1.94 (1.03, 3.70); **.041**				
Past cleanings (≥2 in past year)	31–50 vs. <30	3.64 (1.23, 10.82); **.020**	Male vs. Female	1.09 (0.65, 1.84); .7325	White vs. Black	1.16 (0.67, 2.01); .6027
	>50 vs. <30	4.74 (1.63,13.83); **.0043**				
Daily mouthwash use	31–50 vs. <30	1.50 (0.86, 2.61); .1567	Female vs. Male	1.13 (0.73, 1.69); .6147	Black vs. White	2.01 (1.28, 3.16); **.0025**
	>50 vs. <30	1.26 (0.72, 2.19); .4142				
Frequent dry mouth	31–50 vs. <30	1.22 (0.64, 2.33); .5466	Female vs. Male	1.75 (1.13, 2.71); **.0117**	White vs. Black	1.52 (0.94, 2.48); .0908
	>50 vs. <30	1.18 (0.62, 2.24); .6242				
Carbonated drinks	>50 vs. <30	1.94 (1.07, 3.51); **.0296**	Male vs. Female	1.44 (0.89, 2.33); .1351	White vs. Black	1.17 (0.69, 1.96); .5655
	31–50 vs. <30	2.17 (1.21, 3.91); **.0098**				
Coffee consumption	31–50 vs. <30	1.53 (0.87, 2.68); .1390	Female vs. Male	1.08 (0.71, 1.63); .7156	White vs. Black	1.61 (1.01, 2.59); **.0477**
	>50 vs. <30	1.96 (1.12, 3.42); **.0190**				
Tea consumption	31–50 vs. <30	1.49 (0.86, 2.59); .1541	Female vs. Male	1.14 (0.75, 1.72); .5399	Black vs. White	1.17 (0.75, 1.84); .4898
	>50 vs. <30	1.91 (1.10, 3.31); **.0215**				
Sports drinks	31–50 vs. <30	1.48 (0.83, 2.61); .1806	Male vs. Female	1.23 (0.82, 1.63); .3263	Black vs. White	1.11 (0.71, 1.74); .6333
	>50 vs. <30	2.11 (1.20, 3.72); **.0095**				
Energy drinks	31–50 vs. <30	1.36 (0.69, 2.65); .3737	Male vs. Female	1.46 (0.89, 2.40); .1332	White vs. Black	1.69 (1.04, 2.75); **.0346**
	>50 vs. <30	1.36 (0.70, 2.65); .3708				
Daily alcohol use	<30 vs. 31–50	1.24 (0.69, 2.23); .4817	Male vs. Female	1.79 (1.12, 2.85); **.0148**	Black vs. White	5.15 (2.64, 10.04); **<.0001**
	<30 vs. >50	1.14 (0.64, 2.04); .6480				
Weekly alcohol use	<30 vs. 31–50	1.63 (0.98, 2.69); .0579	Male vs. Female	1.33 (0.91, 1.95); .1453	Black vs. White	2.11 (1.35, 3.32); **.0011**
	<30 vs. >50	1.41 (0.85, 2.32); .1748				
Current smoker	31–50 vs. <30	1.79 (1.03, 3.11); **.0393**	Male vs. Female	1.16 (0.77, 1.74); .4714	White vs. Black	1.22 (0.78, 1.90); .3789
	31–50 vs. >50	1.69 (1.13, 2.54); **.0111**				
Ever a smoker	31–50 vs. <30	1.66 (0.92, 3.01); .0945	Male vs. Female	1.07 (0.69, 1.66); .7579	White vs. Black	1.40 (0.84, 2.32); .1999
	>50 vs. <30	1.29 (0.72, 2.29); .3927				
Oral sex	<30 vs. 31–50	2.05 (1.17, 3.60); **.0126**	Male vs. Female	1.65 (1.02, 2.66); **.0421**	White vs. Black	1.72 (1.06, 2.80); **.0290**
	<30 vs. >50	5.57 (3.06, 10.15); **<.0001**				
	31–50 vs. >50	2.72 (1.69, 4.38); **<.0001**				
Illicit drug use	<30 vs. 31–50	1.07 (0.43, 2.69); .8835	Male vs. Female	1.94 (0.93, 4.06); .0765	White vs. Black	2.25 (1.04, 4.87); **.0391**
	<30 vs. >50	1.15 (0.45, 2.94); .7718				
Marijuana	<30 vs. 31–50	1.61 (0.62, 4.15); .3275	Male vs. Female	1.74 (0.77, 3.93); .1845	White vs. Black	1.96 (0.88, 4.36); .0977
	<30 vs. >50	2.38 (0.88, 6.43); .0875				

*Note*: Bold numbers represent statistically significant *p*‐values.

^a^ The second level of each covariate appearing under “Comparison” indicates the reference group.

^b^ Adjusted odds ratio (aOR); 95% confidence interval [CI] for aOR presented as (upper bound, lower bound); *p* value for testing *H*_0_: aOR = 1 versus *H*_1_: aOR ≠ 1 obtained from multiple logistic regression model including age group, race group, and sex as covariates.

^c^ Brushing and flossing frequency were positively associated [aOR = 13.14 (3.07, 55.95); *p* = .0005].

For nonalcoholic drink consumption, the youngest age group (<30 years) reported lower consumption of carbonated beverages than either those aged 30–50 years or those aged >50 years. For coffee consumption, the youngest age group (<30 years) also reported lower consumption than those aged >50 years, while Whites reported higher coffee consumption when compared with Blacks. For sports drink consumption, the youngest age group (<30 years) once again reported a lower frequency of consumption than those aged >50 years. Finally, Whites consumed energy drinks more frequently than Blacks, and those individuals ≤30 years consumed tea less frequently than those >50 years.

When cigarette smoking was considered, the frequency of current smokers was greater in the 31–50 years age group as compared to those ≤30 years (*p* = .0393) and those persons >50 years. In addition, age was also positively associated with current smoking duration while Whites were found to smoke for a longer duration than Blacks. In contrast, there was no association between age and ever having smoked.

With regard to alcohol consumption, Whites reported less frequent daily or weekly alcohol use as compared with Blacks, and females reported less daily alcohol use than males.

While there was no effect of HIV status on oral sexual encounters, the multivariable model for oral sexual encounters revealed significant associations with age, race, and gender. Individuals ≤30 years of age were more likely than those in 31–50 years to engage in oral sexual behavior. Similarly, individuals 31–50 years were more likely to engage in oral sexual behaviors than individuals >50. Whites reported a higher frequency of oral sexual encounters than Blacks and males reported a higher frequency than females.

HIV− individuals were more likely to use illicit drugs and marijuana, than HIV+ individuals. Moreover, Whites were more likely to use illicit drugs than Blacks.

A geographically similar cohort was studied by our research group between the years of 1998 and 2004. This group included 193 HIV+ persons with the objective of evaluating oral host defense properties in those with and without OPC (Mercante et al., 2006; Quimby et al., 2012). Similar demographic and behavioral data were collected in the prior cohort, including age, race, sex, sexual encounters, oral sex encounters, injection drug use, cigarette smoking, oral hygiene, and dry mouth (Table 3). Unfortunately, alcohol consumption data were not collected in the previous cohort and could not be analyzed further.

**Table 3:** Comparison of demographic and behavioral characteristics of HIV+ study participants in the previous and contemporary cohorts.

	Previous cohort		Contemporary cohort		*p* Value*
	*N*	Percent	*N*	Percent	
	193	32.7	398	67.3	
Categorical variables					
Age group					**<.001**
18–30	32	16.6	52	13.1	.251
31–50	139	72.0	150	37.7	**<.001**
>50	22	11.4	196	49.2	**<.001**
Sex					**.001**
Female	82	42.5	115	28.9	
Male	111	57.5	283	71.1	
Race/ethnicity					**.007**
White	57	29.5	73	18.3	**.002**
Black	130	67.4	306	76.9	**.014**
Other	6	3.1	19	4.8	.346
Current smoker	81	55.9	196	49.8	.208
Daily brushing	32/35	91.4	353	88.9	.647
Never brush	2/35	5.7	24	6.1	.929
Daily flossing	15/33	45.5	138	35.5	.252
Daily mouthwash use	27/35	77.1	229	77.9	.920
Frequent dry mouth	17/35	48.6	118	30.2	**.026**
Illicit drug use	36	22.8	23	27.4	.428
OPC	87	45.1	44	11.1	**<.001**
Antibiotics	21/34	61.8	61	16.8	**<.001**
Antifungals	70/189	37.0	40	11.3	**<.001**

	Mean	SD	Mean	SD	
Continuous variables					
Age	39.2	8.2	47.4	12.0	**<.001**
Pack years	8.4 (*n* = 135)	11.7	22.3 (*n* = 77)	18.5	**<.001**
Blood CD4 cells/µL^a^	229	244	507	324	**<.0001**
	Median	P25, P75	Median	P25, P75	
	140	41, 353	454	263, 674	**<.0001**

*Note*: Bold numbers represent statistically significant *p*‐values.

Abbreviation: OPC, oropharyngeal candidiasis.

^a^ Means and medians were compared using the Student's *t* test and Wilcoxon's test, respectively, due to possible skewness in the distribution.

*p Values are obtained from *χ* ^2^ tests of heterogeneity, except for variables, Never Brush, OPC, and Oral Warts that used Fisher's Exact test.

The contemporary HIV+ cohort is significantly older than the previous HIV+ cohort (median 48 vs. 39 years). Logistic regression analysis showed the contemporary cohort had reduced sexual encounters and injection drug use/illicit drug use compared to the previous cohort (Table 3). Multivariable analyses showed these trends across all age groups, sex, and race. Cigarette smoking was similar between the two cohorts when comparing age, sex, and race. The only exception was reduced smoking in those >50 years of age in the current cohort. No differences were found for oral hygiene behaviors among the two HIV+ cohorts, which included brushing frequency, flossing frequency, and mouthwash use/frequency. Other parameters collected show that HIV+ persons in the current cohort had less OPC, less antifungal use, higher blood CD4 cell counts, and a lower incidence of xerostomia, across age, race, and sex, compared to the previous HIV+ cohort (Table 3).

## 4 DISCUSSION

The initial analysis of survey data for the microbiome study helped confirm that most oral hygiene and recreational behaviors were similar between the HIV+ and HIV− populations implying reasonably balanced cohorts (Griffen et al., 2019). The intent was to enroll a high‐risk HIV− population so that adequate comparisons could be made for the oral microbiome analyses. This analysis expanded on the original study by using the full range of responses to conduct multivariable analyses to identify differences for age, race, and sex in addition to HIV status, and finally to assess changes in parameters over time by comparisons to our previous HIV+ cohort (Leigh et al., 2006; McNulty et al., 2005; Mercante et al., 2006; Quimby et al., 2012).

A notable difference in demographics between the enrollees of the contemporary cohort was the older average age for HIV+ persons (47 vs. 40 years), as well as the dichotomous race relationship with a higher percentage of Blacks in the HIV+ group and higher percentage of Whites in the HIV− group. While race did not appear to play a large role in any differences, the age difference is a likely explanation for several of the differences in oral hygiene and recreational behaviors, including teeth cleanings, brushing, flossing frequency, and duration of cigarette smoking. For teeth cleanings, HIV+ persons had significantly more annual cleanings compared to the HIV− population. The overall older age and increased quality of life in the HIV+ population with current ART (Aragones‐Lopez et al., 2012; Aversa et al., 1998; Bor et al., 2013; Price et al., 2016) may indicate that these HIV+ individuals are more invested in seeking oral healthcare and following the recommended regimen for follow‐up dental visits. In addition, HIV+ subjects under care have better access to free dental care with a dedicated dental clinic located in the same building as their primary care providers. For flossing, while no overt effect of HIV status, the higher frequency of older persons flossing is again likely due to increased investment in oral health. The lower frequency of brushing in the HIV+ population may be a reflection of “dry mouth syndrome” or xerostomia that is not uncommon in HIV+ persons on ART (Diz Dios & Scully, 2014; Nizamuddin et al., 2018). Interestingly, a considerable number of HIV+ individuals reported not brushing at all (6.1%). This may be a sign that brushing is painful when experiencing xerostomia. Relatedly, the higher frequency of mouthwash use in the HIV+ group may be indicative of mouthwash serving as a less painful alternative. But overall there appears to be good commitment to oral hygiene in the HIV+ population.

Similarly, strong oral hygiene behaviors were reported as part of a retrospective study on dental implant success in a cohort of PLWH on ART (*n* = 67; average age of 58 years) (Rubinstein et al., 2019). In that cohort, 77% brushed > once/day and 82% flossed > than once/day. Interestingly, 46% of those enrolled had a history of periodontal disease. The enhanced oral hygiene of that cohort compared to the current cohort (~50% brushing > once/day and ~18% flossing > once/day) may have been from their continued dental treatment for periodontal disease. This would have included oral hygiene instruction as an integral part of periodontal therapy.

While diet and most nonalcoholic beverage consumption were not influenced by HIV status, HIV− persons consumed more nonalcohol carbonated beverages, coffee, and energy drinks than HIV+ persons. In multivariable analyses it was generally the mid‐level (age 30–50) and older (>50 years) age groups who consumed more of these beverages. Any differences in nonalcoholic beverages did not appear to be replaced by more alcohol consumption as both daily and weekly alcohol consumption was similar across HIV status and age. In fact, alcohol consumption was surprisingly low (<50%) for a cohort with high‐risk social behaviors. The only difference noted for alcohol use was a lower consumption in Whites (daily and weekly) and females (daily) compared to their racial counterparts. Historically, PWLH had rates of alcohol consumption as high as 64% (Bing et al., 2001; Dawson‐Rose et al., 2017). The decrease in consumption may reflect age, education, and healthcare provider's influence over time.

Cigarette smoking was very similar between HIV− and HIV+ subjects, both “ever smoked” or “currently smoke.” The only differences noted were a higher frequency of current smoking in the middle age group, and a longer duration of smoking by Whites across all age groups. Interestingly, the overall frequency of smoking (50%) was lower compared to past studies that reported rates in the 60%–75% range (Dawson‐Rose et al., 2017; Pacek & Cioe, 2015). This may be due to the overall decreased prevalence of smoking in the current environment due to public awareness programs, educational programs, and the many negative health consequences associated with smoking. In addition, for PLWH this may also be reflective of an improved quality of life and an investment in their health. A previous cross‐sectional study in China inclusive of recreational/social behavior information on an entirely HIV+ population also observed fairly low levels of cigarette smoking and alcohol use (smoking at 38.8% and alcohol use at 33.8% [Xu et al., 2020]). In both cohorts, a positive correlation was identified between smoking and alcohol consumption such that the two behaviors occur together despite the overall lower frequency of both. As with all surveys, it is possible that the higher percentage of negative responses to smoking and alcohol consumption could be attributed to individuals not being completely truthful in reporting. However, we do not think this is a concern, since responses regarding sexual encounters, illicit drug use, and marijuana use in the present cohort often indicated substantial activity. Accordingly, there were several differences noted for oral sexual encounters, illicit drug use, and marijuana use in the current cohort. Younger White males engaged more often in oral sexual encounters and use marijuana more often. This may be reflective of a generational trend. The fact that HIV− subjects were more likely to use illicit drugs and marijuana is possibly a reflection of both the imbalance of Whites in the HIV− group, as well as an overall decrease in high‐risk behavior that may have decreased with age in the HIV+ population.

The differences noted between the contemporary HIV+ cohort and the previous cohort from 1998 to 2004 (Mercante et al., 2006; Quimby et al., 2012) were likely a direct reflection of the older median age (~10 years) of the contemporary cohort and also influenced by compliance to ART regimens that has led to increased lifespan and improved quality of life. The reduction in both sexual encounters and injection/illegal drug use in the contemporary cohort would also be a reflection of their better quality of life and less risky behavior compared to the previous cohort. Interestingly, although current cigarette smoking was relatively low in the entire contemporary cohort, it remained similar to that in the previous cohort. Hence, it appears that smoking trends have continued over time despite efforts to further reduce smoking through antismoking campaigns or smoking cessation programs. Unfortunately, alcohol consumption could not be compared since those data were not collected in the previous cohort. However, alcohol use was relatively low in the contemporary cohort (<50%). It would have been interesting to evaluate alcohol consumption over time in the two HIV+ cohorts. Interestingly as well were similar oral hygiene activities between the previous and contemporary cohorts despite the age difference. This was in contrast to the differences for similar parameters between the HIV+ and HIV− persons in the contemporary cohort that also had a similar age difference. This may be explained again by HIV+ subjects having free access to dental clinics in HIV outpatient programs. The lack of differences may also be due to comparisons being restricted to only the smaller longitudinal cohort (*n* = 35) that was the only one to collect data on oral hygiene parameters. But suffice it to say that oral hygiene behaviors have been and continue to be strong in HIV+ persons. The prevalence of OPC and associated antifungal use was significantly lower in the contemporary cohort consistent with the reduction in OPC in the advent of improved ART regimens that also increase CD4 cell counts (El Howati & Tappuni, 2018; Tappuni, 2020). Indeed, the CD4 cell counts were significantly higher in the contemporary cohort (median 454 vs. 140 cells/µL) and above the historical threshold for increased susceptibility to opportunistic infections (<200 cells/µL). Finally, xerostomia was also lower in the contemporary cohort which likely reflects a reduction in OPC due to the early initiation of improved ART regimens.

The primary strength of the study is the large size of the cohorts that is not confounded by the geographical location that allowed for comparisons between the HIV+ and HIV− cohorts and the archived HIV+ cohort. Additionally, the HIV− cohort with high‐risk exposure to HIV enabled strong comparisons that are not always accessible for HIV+/− analyses. Limitations of this study include sample size for some parameters from the previous HIV+ cohort, and lack of power estimates for these analyses. Other limitations included potential bias introduced from subject matching in the primary study design, collection of data that was conducted with different personnel as part of the study teams, and omission of unknown confounders via variables not measured and included in our models. But overall, we conclude that the HIV+ population is aging with relatively good oral health and participating in less risky behaviors, likely due to good compliance to current ART regimens and improved quality of life.

## AUTHOR CONTRIBUTIONS

**Donald E. Mercante**: Statistical analysis; database management; preparation of the manuscript. **Emily Guarisco**: Data analysis; graphic illustrations; preparation of the manuscript. **Elizabeth A. Lilly**: Data analysis; data collection; graphic illustrations. **Arni Rao**: Statistical analysis. **Kelly Treas**: Subject recruitment; data collection. **Clifford J. Beall**: Data collection; database management; data analysis. **Zach Thompson**: Data collection; database management; data analysis; statistical analysis. **Ann L. Griffen**: Co‐PI; data collection; database management; data analysis. **Eugene J. Leys**: Co‐PI; data collection; data analysis; **Jose A. Vazquez**: Co‐Inv; subject recruitment; data collection. **Michael E. Hagensee**: Co‐PI; subject recruitment; data collection; data analysis. **Paul L. Fidel**: PI; conception of the manuscript; data collection; data analysis.

## CONFLICT OF INTEREST STATEMENT

The authors have no conflict of interest.

## Data Availability

The data that support the findings of this study are available from the corresponding author upon reasonable request.
